# Leidenfrost droplet trampolining

**DOI:** 10.1038/s41467-021-21981-z

**Published:** 2021-03-19

**Authors:** Gustav Graeber, Kartik Regulagadda, Pascal Hodel, Christian Küttel, Dominic Landolf, Thomas M. Schutzius, Dimos Poulikakos

**Affiliations:** 1 Laboratory of Thermodynamics in Emerging Technologies, Department of Mechanical and Process Engineering, ETH Zurich, Zurich, Switzerland; 2 Present Address: Laboratory for Multiphase Thermofluidics and Surface Nanoengineering, Department of Mechanical and Process Engineering, ETH Zurich, Zurich, Switzerland

**Keywords:** Surfaces, interfaces and thin films, Wetting, Fluid dynamics, Phase transitions and critical phenomena

## Abstract

A liquid droplet dispensed over a sufficiently hot surface does not make contact but instead hovers on a cushion of its own self-generated vapor. Since its discovery in 1756, this so-called Leidenfrost effect has been intensively studied. Here we report a remarkable self-propulsion mechanism of Leidenfrost droplets against gravity, that we term Leidenfrost droplet trampolining. Leidenfrost droplets gently deposited on fully rigid surfaces experience self-induced spontaneous oscillations and start to gradually bounce from an initial resting altitude to increasing heights, thereby violating the traditionally accepted Leidenfrost equilibrium. We found that the continuously draining vapor cushion initiates and fuels Leidenfrost trampolining by inducing ripples on the droplet bottom surface, which translate into pressure oscillations and induce self-sustained periodic vertical droplet bouncing over a broad range of experimental conditions.

## Abstract (web summary)

The classic Leidenfrost phenomenon is familiar, yet its physics is rather complex. Graeber et al. observe the unexpected development of repeated hopping of a droplet trampolining on its own vapor cushion on a hot plate and show under which conditions this self-initiated motion occurs.

## Introduction

Interactions between vaporizing liquid droplets and solid surfaces are ubiquitous in nature and everyday life, and critically affect the safety and performance in a variety of technical disciplines, ranging from energy conversion (e.g., boiling^1^ and spray cooling^2^), to industrial production (e.g., additive manufacturing^3^ and coatings^4^) and medical technology (e.g., microfluidics^5^ and lab on a chip^6^). One of the most fascinating dewetting phenomena that arise in some of these applications is the Leidenfrost effect. In 1756, Johann Gottlob Leidenfrost discovered that water droplets placed on very hot surfaces do not boil, but instead hover on a cushion of their own vapor and slowly evaporate^7,8^. Intensive research in the past centuries revealed that the Leidenfrost effect is not limited to the classical case of a water droplet on a hot rigid surface. In fact, it occurs on hot and cold surfaces and for a variety of liquids, surface materials and textures, whenever a stable, insulating gas layer separates the volatile liquid from the underlying substrate^9–18^. In these numerous manifestations, it has been shown that the Leidenfrost effect can reduce the heat transfer between droplet and substrate with significant negative consequences in all related applications (e.g., spray cooling^2^ and nuclear reactors^19^), but that it can also e.g., provide controlled droplet motion^16,17^, reduce fluidic drag^20^, or enhance chemical reactions^21^. Therefore, its appearance and development with time must be fully understood. Currently, it is widely accepted that the droplet and the vapor co-exist in a quasi-static Leidenfrost equilibrium, which determines the droplet shape and the vapor cushion thickness based on a balance between gravity, surface tension, and lifting force resulting from the draining vapor^22–24^.

In contrast, here we present the manifestation of self-initiated and sustained Leidenfrost droplet trampolining, a clear deviation from the traditionally accepted quasi-equilibrium state of Leidenfrost droplets. We unravel the physical reasons behind this self-propulsion mechanism by modeling the Leidenfrost droplet via a mass-spring-damper system and exploring the effect of three intertwined governing parameters: The Bond number, Bo, which rationalizes when surface tension can overcome gravity; the capillary number, Ca*, which elucidates the conditions where intervening vapor flows near the surface can initiate ripples at the bottom of the droplet; and the Ohnesorge number, Oh, which describes the importance of liquid viscosity affecting the ability of the droplet to perform the underdamped motion. We show how these parameters both explain the manifestation of Leidenfrost trampolining and define the parametric domain where it occurs.

## Results and discussion

### Vertical self-propulsion

**Fig. 1:**
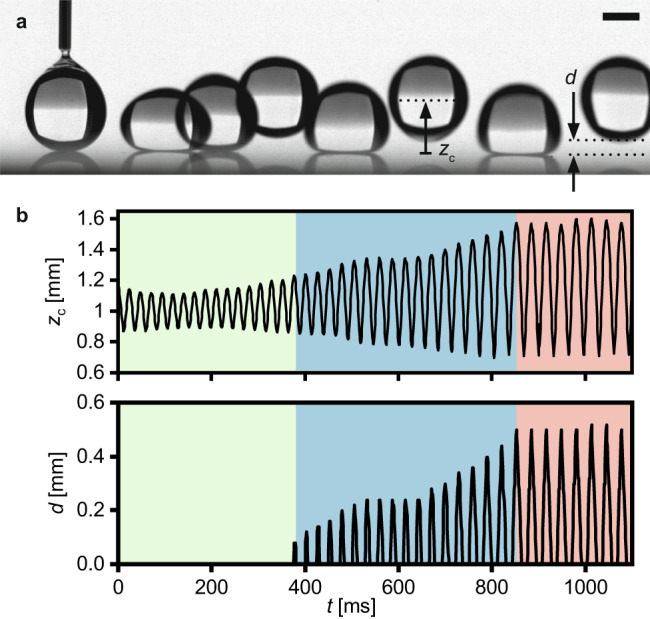
Leidenfrost droplet trampolining. **a** Side-view chronophotographic image of a water droplet gently deposited on a flat silicon wafer heated to a temperature of *T*_S_ = 251 °C. For the case in (**a**), we quantify the droplet motion and show in (**b**) plots of its centroid position, *z*_c_, and the visible vapor cushion thickness between the droplet and the heated surface, *d*, vs. time, *t*, respectively. After deposition, the droplet is in a Leidenfrost state and *z*_c_ oscillates with increasing amplitude (green section in (**b**)). After a few oscillations, the droplet performs its first jump so that a visible gap between the droplet and the heated surface appears. During the successive jumps, the droplet gains height, and the maximum gap height during a jump increases (blue section in (**b**)), until the droplet reaches jumping heights of 0.5 mm (red section in (**b**)). Over the course of the observation, the droplet slowly moves laterally on the surface (Supplementary Movie 1). Time-zero is when the droplet detaches from the needle. The time steps of the chronophotography in (**a**) are 600 ms between the first and the second frame and 80 ms for all subsequent frames. The initial droplet radius is 1.1 mm. The scale bar represents 1 mm. Source data are provided as a Source Data file.

Figure 1 shows the Leidenfrost trampolining phenomenon that we observe with high-speed imaging after gently placing a water droplet (initial radius *r*_i_ = 1.1 mm) on a smooth silicon surface heated to a temperature of *T*_S_ = 251 °C (Supplementary Fig. 1 shows the experimental setup). After deposition, the droplet is in a Leidenfrost state and its centroid position, *z*_c_, oscillates with an increasing amplitude as a function of time, *t*. After a few underdamped oscillations in place, the droplet performs its first jump in which the current Leidenfrost vapor cushion thickness, *d*, substantially exceeds its equilibrium value of *d*_0_ ≈ 0.02 mm for a droplet of this size^13^ (Fig. 1b). During the subsequent successive jumps, the maximum vapor cushion thickness during a jump, *d*_max_, increases, until it plateaus at 0.5 mm (Supplementary Movie 1). Our observations of Leidenfrost droplets gaining in jump height are intriguing as the jumping far exceeds unavoidable initial small droplet oscillations caused by the gentle deposition process, and as it stands in contrast to the existing literature on the impact of Leidenfrost droplet on solid surfaces, reporting decreasing rebound heights after impact^25–29^. Although single bounce-off phenomena have occasionally been reported in the Leidenfrost effect literature^13^, we will show that the repeated, regular trampolining motion presented here must be treated systematically, as a phenomenology of its own (see Supplementary Note 1: Other Leidenfrost Studies).

### Droplet bond number

**Fig. 2:**
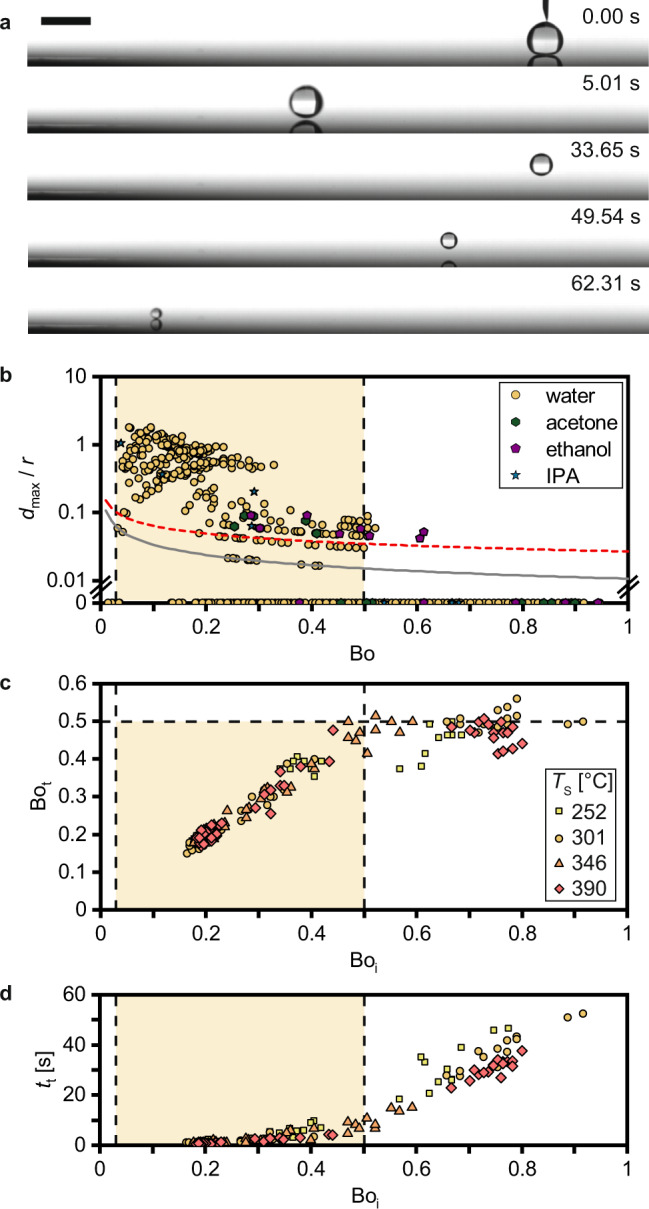
The effect of droplet Bond number on Leidenfrost trampolining. Liquid droplets gently deposited on a concave silicon wafer heated above the Leidenfrost temperature. **a** Long-term observation of a water droplet with an initial Bond number, Bo_i_ = 0.3 (initial droplet radius, *r*_i_ = 1.4 mm), and surface temperature, *T*_S_ = 302 °C. Time-zero is when the droplet detaches from the needle (Supplementary Movie 2). The scale bar represents 4 mm. **b** Maximum gap height during a jump divided by the instantaneous droplet radius, *d*_max_/*r*, as a function of the Bond number, Bo, at *T*_S_ = 301 ± 3 °C extracted from *n* = 70 independent experiments for water (yellow circle), acetone (green hexagon), ethanol (purple pentagon) and isopropyl alcohol (IPA, blue star), see Supplementary Movie 3. The red dashed line represents the equilibrium vapor layer thickness *d*_0_ (computed for water and *T*_S_ = 300 °C, see Supplementary Text on Leidenfrost Modeling), and the gray line is the lower limit of optical resolution in our experiments. Data points at *d*_max_/*r* = 0 represent non-trampolining droplets. **c**, **d** Droplet Bond number at the first trampolining jump, Bo_t_, and time passing until the start of Leidenfrost trampolining, *t*_t_, vs. Bo_i_ for different *T*_S_ measured for *n* = 215 independent experiments. 90% of the experiments showed Leidenfrost trampolining. The legend shows the mean for *T*_S_ with a standard deviation of ±3 °C around the mean. Source data are provided as a Source Data file.

First, we investigated the macroscopic motion of the droplet and the importance of gravity vs. surface tension, as expressed by the Bond number, defined as Bo = Δ*ρgr*^2^*γ*^−1^, where Δ*ρ* is the density difference between the liquid, *ρ*_l_, and the surrounding vapor, *ρ*_v_, *g* is the gravitational constant, and *γ* is the surface tension between liquid and vapor. Figure 2a shows a long-term observation of a water droplet deposited on a slightly concave silicon wafer heated to *T*_S_ = 302 °C. After deposition at *t* = 0 s, the droplet (initial Bond number Bo_i_ = 0.3) hovers in a Leidenfrost state, oscillates with increasing amplitude around the altitude of its center of gravity until it performs its first jump (*t* ≈ 5 s), and continues to trampoline for ≈50 s (Supplementary Movie 2), simultaneously reducing its instantaneous radius, *r*, and with it Bo due to evaporative mass loss. After approximately 60 s, Bo decreased by an order of magnitude reaching ≈0.04 and the droplet stops bouncing. From 70 independent experiments similar to the one shown in Fig. 2a, we plot *d*_max_/*r* vs. Bo in Fig. 2b illustrating the critical influence of Bo on Leidenfrost trampolining for the four tested liquids—water, acetone, ethanol, and isopropyl alcohol (Supplementary Movie 3). Beyond an upper limit of Bo > 0.5 droplets do not trampoline, while droplets that evaporate below a lower limit of Bo < 0.04 stop bouncing. This delineates a favorable region of Bo where Leidenfrost trampolining is initiated and sustained.

The long-term observations allow investigating the critical aspect of trampolining initiation as presented in Fig. 2c, d. For surface temperatures *T*_S_ between 252 and 390 °C, we varied the initial Bond number, Bo_i_, and measured the Bond number at the moment of spontaneous initiation of trampolining, Bo_t_, as well as the time between droplet deposition and the start of trampolining, *t*_t_. When placing a droplet with Bo_i_ > 0.5, it does not start trampolining immediately, but instead hovers above the surface, oscillates uncoordinated in place, and gradually evaporates. Once the droplet reaches Bo ≈ 0.5, trampolining is initiated. Large droplets with Bo_i_ → 1 take more than 30 s of evaporation time before they finally perform their first jump. In contrast, when placing droplets with Bo_i_ < 0.5, trampolining starts with substantially less delay. The observations of droplets with large Bo_i_ and long *t*_t_ underpin that Leidenfrost trampolining can be initiated by the droplet itself without the necessity of external input. In fact, all momentum transferred to the droplet during careful deposition is dissipated after a time of 30 s. Interestingly, the results in Fig. 2c, d do not depend on *T*_S_. From this and further experiments (see Supplementary Fig. 2 and Movie 4), we conclude that *T*_S_ has to be sufficient for the droplet to reach a Leidenfrost condition, while a further increase does not alter the trampolining dynamics (see Supplementary Note 2: Surface Temperature Effect).

### Trampolining mechanism

**Fig. 3:**
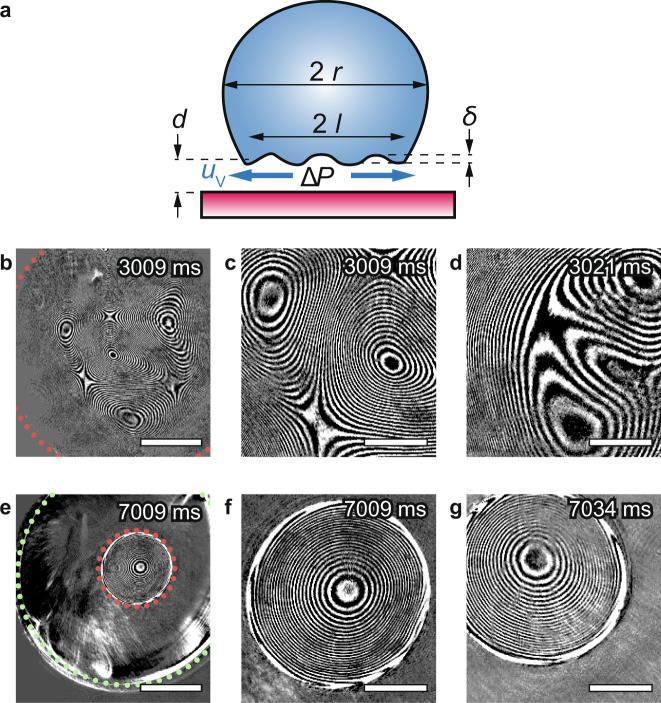
Leidenfrost trampolining mechanism. **a** Sketch of a Leidenfrost droplet of instantaneous radius, *r*, vapor pocket radius, *l*, and average vapor cushion thickness, *d*, hovering above a hot surface due to an average overpressure under the droplet, Δ*P*. The vapor drains under the droplet (illustrated by blue arrows) with a vapor velocity, *u*_v_, and causes ripples of amplitude, *δ*. Bottom-view interferometric images of relatively (**b**–**d**) large (not trampolining, Bond number Bo → 1) and (**e**–**g**) intermediate-sized (trampolining; Bo = 0.13; *r* = 0.9 mm, green dotted line) Leidenfrost droplets on a heated sapphire plate (**b**–**d**: *T*_S_ = 280 °C; **e**–**g**: *T*_S_ = 250 °C). The vapor pocket radii, *l*, in **b** (*l* = 1.1 mm) and **e** (*l* = 0.25 mm) are shown by a red dashed line. **c** Magnified image of **b** showing the interference patterns resulting from the complex shape of the large vapor pocket and **d** its shape 12 ms later, revealing its oscillatory behavior despite the fact that it is not trampolining. **f** Magnified image of **e** revealing the symmetric shape of the small, dome-shaped vapor pocket and **g** its shape 25 ms later revealing its oscillatory behavior while remaining relatively axisymmetric. Time-zero: **b**–**d**, the start of droplet observation; **e**–**g**, the moment the droplet is placed on the heated surface. Supplementary material: **b**–**d**, Supplementary Fig. 8 and Movie 4; **e**–**g** Supplementary Fig. 9 and Movie 5. Scale bars: **b** and **e** 0.5 mm; **c**, **d**, **f**, and **g**: 0.2 mm.

To understand how the droplet can self-initiate and sustain trampolining, we combine Leidenfrost droplet modeling with interferometric microscopy observations of the Leidenfrost vapor cushion (Fig. 3a). We observe experimentally that the liquid–vapor interface under both large (Bo → 1, Fig. 3b–d) and intermediate-sized Leidenfrost droplets (Bo = 0.13, Fig. 3e–g) shows a transient topography with pronounced ripples^13^. As we will see below, these ripples are a critical requirement for trampolining initiation.

The Leidenfrost system consists of a denser fluid (the liquid water droplet) located on top of a lighter, draining fluid (the vapor cushion) in a gravity field—a configuration in principle prone to being unstable. Simultaneously, the droplet is continuously exposed to a variety of disturbances, since it is impossible to shield a real physical system from small perturbations^30^. To understand how these perturbations can disturb the Leidenfrost equilibrium we perform Leidenfrost droplet modeling following a simplified lubrication analysis given in ref. ^24^, assuming that the vapor draining under the droplet behaves like a quasi-steady, quasi-developed incompressible flow in a one-dimensional channel with channel height *d*_0_ and channel length 0.81 *r* (see Supplementary Note 3: Leidenfrost Modeling and Supplementary Fig. 3). Based on this model, the overpressure under the Leidenfrost droplet (above the environmental pressure) is Δ*P* = 2.6 *µ*_v_*a*_v_Ja *r*^2^ (1 + 0.2 Ja Pr^−1^) *d*_0_^−4^. Here, *µ*_v_ is the dynamic viscosity of vapor, *a*_v_ = *κ*_v_*ρ*_v_^−1^*c*_*P*,v_^−1^ is the thermal diffusivity of vapor, with *κ*_v_, *ρ*_v_, and *c*_*P*,v_ being the thermal conductivity, the density, and the isobaric heat capacity, respectively. The Prandtl number is defined as Pr = *c*_*P*,v_ *µ*_v_*κ*_v_^−1^ and the Jakob number, which relates sensible to latent heat, is defined as Ja =  *c*_*P*,v_ (*T*_S_ − *T*_L_) *H*_lv_^−1^, where *T*_L_ is the average temperature of the droplet and *H*_lv_ is the enthalpy of vaporization. The channel height *d*_0_ is given as *d*_0_ = {1.62 *µ*_v_*a*_v_Ja *r* (1 + 0.2 Ja Pr^−1^)/[*g* (*ρ*_l_ − *ρ*_v_)]}^1/4^. Based on this modeling, we can show that due to the nonlinear dependence between vapor cushion thickness and overpressure under the droplet (Δ*P* ~ *d*^−4^), any volume conserving perturbation superimposed over the equilibrium vapor layer thickness will translate into an increase of the effective overpressure (see Supplementary Note 4: Overpressure Generation and Supplementary Fig. 4). Consequently, the unavoidable perturbations change the effective overpressure under the droplet, disturb the Leidenfrost equilibrium, and provide the initial seed for the observed vertical droplet motion.

To understand when ripples at the bottom of the droplet with amplitudes in the μm-range—as observed experimentally (Fig. 3)—can form under the droplet, we consider the forces acting on the bottom interface of the droplet. The main perturbing force driving towards deformation of the droplet bottom surface results from the draining vapor. It transmits a shear force that acts to deform the interface and create a ripple. The force due to surface tension resists the interface deformation and therefore resists the ripple formation. Whether shear force or surface tension dominates near the interface determines if ripples can be formed underneath the droplet or not—their ratio is typically expressed as a capillary number. To estimate an appropriate capillary number, we employ the lubrication Leidenfrost modeling as introduced above (see Supplementary Note 3: Leidenfrost Modeling). On this basis, we compute a modified capillary number Ca* = Δ*P*_viscous_/Δ*P*_Laplace_, which compares the maximum viscous pressure that the draining vapor exerts at the droplet bottom surface, Δ*P*_viscous_ = 0.81 *τ*_interface_ *r*/*d*_0_, to the Laplace pressure of typical created ripples, Δ*P*_Laplace_ = 2 *γ r*_Laplace_^−1^. Here, *τ*_interface_ = 6 *µ*_v_*u*_v_/*d*_0_ is the maximum shear stress at the interface^24,31^, *u*_v_ is the average exit velocity of the draining vapor when leaving the channel under the droplet given as *u*_v_ = 0.81 *a*_v_Ja*rd*_0_^−2^, and *r*_Laplace_ is the radius of curvature of the ripple. Based on experimental observations (see Fig. 3) we estimate the ripple amplitude to be *d*_0_/4 and the ripple wavelength to be *r*, so that *r*_Laplace_ = 1/8 (*r*^2^/*d*_0_) (see Supplementary Fig. 5). Therefore, Ca* = 0.3 Ca (*r*/*d*_0_)^3^, where the standard capillary number is defined as Ca = *µ*_v_*u*_v_*γ*^−1^. In summary, for given *T*_S_, *µ*_v_, *a*_v_, *ρ*_l_, *ρ*_v_, *γ*, Pr, and Ja, (all constant) and considering the above dependencies (*d*_0_ ~ *r*^1/4^, Ca ~ *u*_v_ ~ *r*^1/2^), we conclude that Ca* only depends on droplet size and is proportional to *r*^2.75^. Inserting appropriate values^32^ for a large water droplet with Bo = 0.6 (*r* = 2 mm) at *T*_S_ = 300 °C, we obtain Ca* = 3 indicating that the shear stress of the draining vapor can locally compete with surface tension to cause ripples at the droplet bottom interface^31^. In contrast, for very small droplets with Bo = 0.04 (*r* = 0.5 mm) at *T*_S_ = 300 °C, Ca* = 0.1 indicating that the shear stress is negligible compared to surface tension, since Ca* ~*r*^2.75^. Consequently, the droplet behaves like a hard-sphere, and the draining vapor cannot cause ripples. Since ripples and related droplet oscillations are required for trampolining initiation, we propose this as an explanation for our observations in Fig. 2, where we showed that for very small droplets (Bo = 0.04) the trampolining action ceases to exist.

In the following, we elucidate how the observed ripples can translate into vertical droplet motion and trampolining. From the interference images in Fig. 3, we estimate the ripple amplitude to be *δ* ≈ 5 µm. Based on the Leidenfrost modeling, we conclude that for *d*_0_ ≈ 0.02 mm (refs. ^13,24^), Δ*P*(*d* = *d*_0_ − *δ*)/Δ*P*(*d* = *d*_0_) = 3.2 when keeping all other variables constant. This quantifies the overpressure to locally expect from the observed ripples. To shed light on how the overpressure can lead to trampolining, we model a trampolining droplet as a mass-spring-damper (MSD) system with two individual masses and an integrated heat transfer model (see Supplementary Note 5: MSD model, Supplementary Figs. 6 and 7). We find that the MSD model captures the main dynamics of Leidenfrost droplet trampolining. From an initial resting position, the model droplet starts to vibrate with increasing amplitude (represented by the oscillatory motion of the centroid position *z*_c_) until it performs its first jump where the vapor cushion thickness *d* exceeds the equilibrium vapor cushion thickness. The amplitude of *z*_c_ (where *z*_c_ varies between 0.7 and 1.3 mm), the magnitude of *d* (reaching values of up to 0.4 mm), as well as the frequency of the vibrations match well with the experimental results given the simplicity of the MSD model (see Supplementary Fig. 7 for a comparison between measured data and simulation). The simulation allows to quantify the magnitude of the pressure force, *F*_p_, acting on the droplet due to the overpressure as compared to the force due to gravity, (*m*_d_ *g*), see Supplementary Fig. 7. We find that the ratio *F*_p_/(*m*_d_ *g*) can briefly exceed values of 50, which illustrates that the pressure force can in fact overcome gravity and propel the droplet away from the hot surface into a trampolining motion.

Both large (Bo = 1) and intermediate-sized Leidenfrost droplets (Bo = 0.13) show pronounced ripples (Fig. 3). However, only the intermediate-sized droplets trampoline (Fig. 2). We find that large droplets are unable to behave like an elastic bouncing ball but instead wobble with multiple uncoordinated modes of macroscopic droplet oscillation dissipating more kinetic energy, which is no longer available to the bouncing motion. This can be observed in side-view imaging (see e.g., Supplementary Movie 2), but especially well in the interference patterns of the vapor cushions (Fig. 3b–d, Supplementary Fig. 8 and Supplementary Movie 5). In contrast, intermediate-sized droplets do not wobble. Instead, they can bounce elastically like a ball. During the elastic impacts, the vapor layer forms a single dome-shaped vapor pocket (Fig. 3e–g, Supplementary Fig. 9 and Supplementary Movie 6). We quantified the number of vapor pockets under trampolining and non-trampolining droplets in Supplementary Fig. 10. In Supplementary Movie 7, we captured the transition from a wobbling, large, non-trampolining droplet, to a ball-like, intermediate-sized trampolining droplet in a continuous experiment of an evaporating droplet. The number of persisting droplet oscillation modes, and therefore the risk to wobble, can be estimated based on the droplet-size-dependent oscillation mode decay time given in refs. ^33,34^ as *t*_*m*_ = *ρ*_l_*r*^2^ [*µ*_l_ (*m*_nbr_ − 1) (2*m*_nbr_ + 1)]^−1^ for freely oscillating droplets, where *µ*_l_ is the liquid viscosity and *m*_nbr_ is an integer representing the oscillation mode number of the various vibration modes of the liquid droplet^33,34^. It shows that in large droplets, oscillations can persist substantially longer and multiple oscillation modes can exist simultaneously (see Supplementary Note 6: Droplet Damping and Supplementary Fig. 11). Hence, large droplets wobble, while intermediate-sized droplets trampoline. This analysis underpins the existence of an upper limit in Bo as reported in Fig. 2. The energy dissipated in large, wobbling droplets can be represented in our MSD model (see Supplementary Note 5: MSD model) as an increase in the damping of the droplet. We find that for a doubled damping coefficient in the MSD system and otherwise unchanged conditions, no trampolining motion occurs.

### Liquid viscosity

**Fig. 4:**
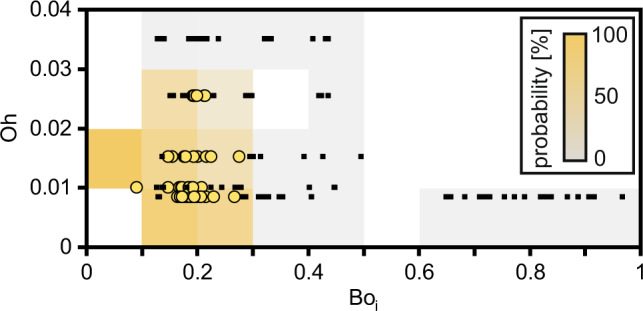
Leidenfrost trampolining occurrence. The occurrence as a function of the initial Bond number, Bo_i_, and Ohnesorge number, Oh, is evaluated 1.4 s after gentle droplet deposition on concave silicon and sapphire substrates heated to *T*_S_ ≈ 300 °C. Yellow circles show experiments where Leidenfrost trampolining was observed, while black squares represent the absence of Leidenfrost trampolining. Shading highlights the occurrence probability. *n* = 154 individual experiments. Source data are provided as a Source Data file.

To explore the effect of liquid viscosity, *µ*_l_, on Leidenfrost trampolining, we performed experiments with aqueous glycerol mixtures. With all other parameters being the same, increasing the droplet viscosity limits the Leidenfrost trampolining manifestation (see Supplementary Fig. 12, Supplementary Movies 8 and 9), which implies that Bo is clearly not the only droplet variable to consider. Figure 4 shows the Leidenfrost trampolining occurrence for varying Bo_i_ and *µ*_l_, as expressed by the Ohnesorge number Oh = *µ*_l_ (*ρ*_l_*γd*_0_)^−0.5^. Since the oscillation mode decay time is inversely proportional to *µ*_l_, for high droplet viscosities, the ripples on the droplet surface and the oscillations of the droplet are no longer underdamped and cannot persist, rendering Leidenfrost trampolining impossible.

Based on our experimental observations, modeling and simulations, we put forth the following explanation for the Leidenfrost trampolining mechanism: For Leidenfrost droplets in an appropriate Bo, Ca*, and Oh domain, the draining vapor causes ripples at the bottom interface of the droplet. The ripples locally reduce the vapor cushion thickness causing a local enhancement of heat transfer and vaporization resulting in an overpressure increase beneath the droplet. The Leidenfrost equilibrium is disturbed, lifting the droplet above the standard equilibrium distance to the hot surface reducing resistance to flow, evaporation rate, and overpressure. Consequently, in a self-correcting action, the droplet falls back towards the hot surface due to gravity, this time further compressing the vapor pocket and allowing the droplet to gain more momentum. This cyclic, underdamped process leads to sufficient momentum gain for macroscopically visible vertical droplet oscillations. After a series of vibrations in place, the droplet detaches from the rigid surface and starts trampolining, markedly shifting its center of gravity upwards.

Leidenfrost trampolining occurs for different liquids such as water, acetone, ethanol, IPA, liquid nitrogen, and aqueous glycerol solutions and on a variety of surfaces including silicon micro-pillar surfaces, hydrophobic silicon, and aluminum (Supplementary Fig. 13, Movies 10–13). We conclude that Leidenfrost droplet trampolining is a robust phenomenon of universal nature, constituting an unexpected manifestation of the 250-year-old famous Leidenfrost effect. Our findings are directly relevant to most situations where Leidenfrost droplets occur such as cooling applications, chemical synthesis, and microfluidics since the trampolining motion affects critical droplet properties including mobility and heat transfer.

## Methods

### Materials

We performed most experiments on smooth, single side mirror-polished silicon wafers of 100 mm diameter, 375 µm or 500 µm in thickness. The silicon wafers were either as-purchased (flat) or treated to be concave. To create the concave silicon wafers resulting in self-centering of the mobile Leidenfrost droplets, we thermally oxidized the silicon wafer on both sides to obtain an oxide layer of approximately 10 µm thickness. Subsequently, we removed the thermal oxide from the mirror-polished wafer top side by buffered hydrogen fluoride. Due to internal stresses within the wafer, it bends to a concave shape with the center being indented by ≈100 µm with respect to the wafer edges. We also used flat, textured silicon wafers decorated with micro-pillars arranged in a regular square array with a pillar diameter of 10 µm, a pillar height of 31 µm, and a center to the center pitch of 100 µm. We fabricated the texture using standard photolithography and dry etching. We also used hydrophobized silicon prepared with fluoro-chemistry wet treatment based on 1H, 1H, 2H, 2H-perfluorodecyltrichlorsilane, 96% (FDTS, 96%, Alfa Aesar). After cleaning the samples in an oxygen plasma, we immersed them in a mixture of 10 µL FDTS in 20 mL hexane for 2 min, followed by rinsing in hexane, rinsing in IPA, rinsing in deionized water, and heating for 10 min on a hotplate at 120 °C (see ref. ^35^ for further details). In addition, we performed experiments on polished aluminum plates, 60 mm in diameter and 10 mm in thickness. The aluminum plates had a slight concave continuous curvature machined on it, giving it the shape of a plano-concave lens with the center being 1 mm indented. For bottom-view visualization, we used a flat sapphire window (thickness 1 mm, diameter 25.4 mm, UQG Ltd.) and a plano-concave sapphire lens (thickness 8.38 mm, diameter 35.1 mm, center indented by 1.38 mm, Guild Optical Associates, Inc.). For the liquid droplets, we used pure deionized water (Merck Milli-Q direct, resistivity > 18.2 MΩ cm), liquid nitrogen, and mixtures of deionized water with glycerol.

### Side-view visualization

For side-view visualization, we gently placed the millimeter-sized droplets on the heated solid surfaces in open air at ambient pressure and temperature using a setup as sketched in Supplementary Fig. 1. If not stated otherwise, gentle deposition means that the droplet did not jump in the first oscillation after detachment from the needle (i.e., *d*_max,first_ ≈ 0 mm below the limit of resolution of typically 0.026 mm). We heated the surface with a hot plate (IKA C-MAG HS 7 digital). We placed water droplets with a syringe pump (Harvard Apparatus Pump 33), liquid nitrogen with a metal spoon, and aqueous glycerol mixtures with a syringe by hand. We recorded side-view images either with a Photron SA 1.1 or a Phantom v9.1 high-speed camera at a frame rate of 500–1000 frames per second using backlight illumination (LED Panel slim 22 W, Swiss Energy Solution AG). On top of the hot plate, we placed a copper heat spreader. The heat spreader temperature *T*_Cu_ was measured using an RTD embedded in the copper block. *T*_Cu_ was used to control the hot plate temperature by the internal hot plate controller. The reference heat spreader temperature *T*_Cu,ref_ was measured using a K-type thermocouple embedded in the copper block. The surface temperature *T*_S_ was measured using a K-type thermocouple surface probe (Omega, 88046K_IEC). The surface reference temperature *T*_S,ref_ was measured by a surface-mountable resistance temperature detector (RTD, IST AG) clamped on the test surface. The difference between *T*_S_ and *T*_S,ref_ was typically around 1 K and always less than 10 K giving an estimate for the measurement uncertainty of *T*_S_ for the side-view visualization setup. We used a micrometer tilting stage under the hot plate to adjust the test surface tilt angle.

### Interference imaging

For bottom-view interference imaging we used a custom-built inverted microscope as shown in Supplementary Fig. 1 based on the system in ref. ^35^. We deposited droplets by hand from a syringe onto a flat sapphire window and a plano-concave lens that was placed on a ring-shaped brass heat spreader and heated using a resistive ring heater (Omega, A-205/240). On the flat window, to prevent droplet lateral movement, we kept the small droplets in contact with the needle during the experiments. We recorded images using a blue laser (Thorlabs CPS405, wavelength 405 nm), a beam splitter (Thorlabs, CM1-BP145B1), a 10× or 4× objective (Olympus, PLN), and the high-speed camera. The number of fringes is multiplied by a quarter of the laser wavelength to compute vertical distances from the recorded images. In the bottom-view visualization setup, *T*_S_ is measured using a surface-mounted T-type thermocouple (Omega, 5SRTC-TT-TI-40-1M).

### Image analysis

We performed image analysis with the Photron Fastcam Viewer software, MATLAB, and ImageJ. We estimated the droplet radius, *r*, as half the average droplet width and height. In Fig. 2b, after deposition of the droplet, the first measurement of *d*_max_ is performed after one second. Subsequently, at a maximum of every 2 s, a measurement is performed as long as the droplet is in focus and until it leaves the area of observation. For bottom-view interference visualization we applied a floating median background subtraction MATLAB code to reduce noise.

### Materials data

The properties of the liquid water droplet are evaluated for saturated water at 100 °C and of the draining water vapor for saturated water vapor at 200 °C (ref. ^32^). For the Bond number, we assume Δ*ρ* = *ρ*_l_. For the Ohnesorge number in Fig. 4, *µ*_l_ is computed at 100 °C according to refs. ^36,37^ and the vapor cushion thickness is set to *d*_0_ = 0.02 mm. The amounts of glycerol added to the water do not substantially affect the boiling point of the mixture^38^.

## Supplementary information

Supplementary Information

Descriptions of Additional Supplementary Files

Supplementary Movie 1

Supplementary Movie 2

Supplementary Movie 3

Supplementary Movie 4

Supplementary Movie 5

Supplementary Movie 6

Supplementary Movie 7

Supplementary Movie 8

Supplementary Movie 9

Supplementary Movie 10

Supplementary Movie 11

Supplementary Movie 12

Supplementary Movie 13

## Data Availability

The data generated and used in this study is provided as a Source Data file and available from OSF.io repository, DOI 10.17605/OSF.IO/ZTX3D. Source data are provided with this paper.

## Source data

Source Data

## Code availability

The code used in this study is available from the corresponding author upon request.
