# Supplementary material for: Leidenfrost droplet trampolining
Source: Nat Commun. 2021 Mar 19;12:1727. doi: 10.1038/s41467-021-21981-z (PMC7979863; doi:10.1038/s41467-021-21981-z)
Supplement: Supplementary file 1 — Supplementary Information [file 41467_2021_21981_MOESM1_ESM.pdf]

Supplementary Information: Leidenfrost Droplet Trampolining

Gustav Graeber[1], Kartik Regulagadda[1], Pascal Hodel[1], Christian Küttel[1], Dominic Landolf[1], Thomas M. Schutzius[1,*], Dimos Poulikakos[1,*]

[1] Laboratory of Thermodynamics in Emerging Technologies, Department of Mechanical and Process Engineering, ETH Zurich, Sonneggstrasse 3, CH-8092 Zurich, Switzerland.

Keywords: Leidenfrost, Vaporization, Droplet, Ripples, Oscillations

[*] To whom correspondence should be addressed.

Prof. Dimos Poulikakos
ETH Zurich
Laboratory of Thermodynamics in Emerging Technologies
Sonneggstrasse 3, ML J 36
CH-8092 Zürich
SWITZERLAND
Phone: +41 44 632 27 38
Fax: +41 44 632 11 76
dpoulikakos@ethz.ch

Thomas M. Schutzius
Current Address:
ETH Zurich
Laboratory for Multiphase Thermofluidics and Surface Nanoengineering
Sonneggstrasse 3, ML J 27.2
CH-8092 Zürich
SWITZERLAND
Phone: +41 44 632 46 04
thomschu@ethz.ch

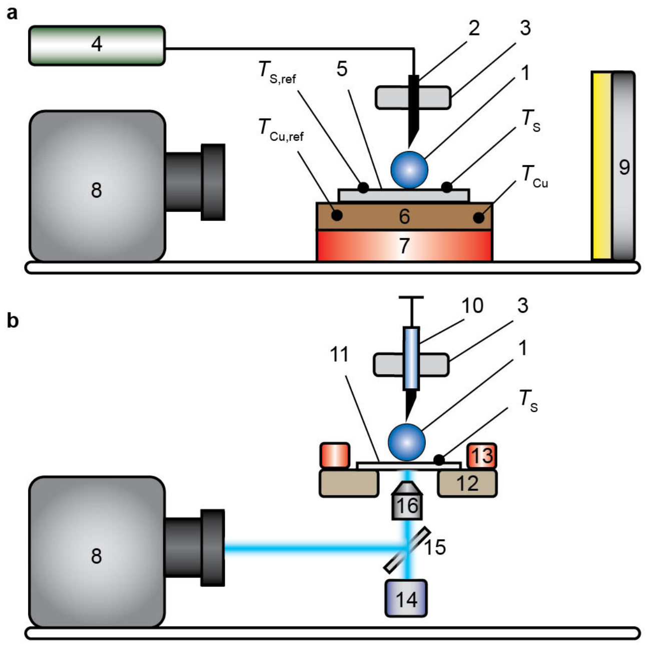

Supplementary Fig. 1 | Experimental setups. a, Setup for side-view visualization: A droplet (1) is gently deposited from a needle (2), which is held by a height-adjustable holder (3) and fed by a syringe pump (4), onto the test surface (5). The test surface is clamped on a copper heat spreader (6) and heated by a hot plate (7). Images are obtained with a high-speed camera (8) using backlight LED illumination (9). The surface temperature T_{S} is measured using a K-type thermocouple surface probe. The surface reference temperature $T_{\mathrm{S,ref}}$ is measured by a surface-mountable resistance temperature detector (RTD) clamped on the test surface. The heat spreader temperature T_{Cu} is measured using an RTD embedded in the copper block. T_{Cu} is used to control the hot plate temperature using the internal temperature controller of the hot plate. The reference heat spreader temperature $T_{\mathrm{Cu,ref}}$ is measured using a K-type thermocouple embedded in the copper block. A micrometer tilting stage is used to adjust the test surface tilt angle. **b**, Setup used for bottom-view visualization. Droplets are deposited by hand from a syringe (10) onto a sapphire window (11) that is placed on a ring-shaped brass heat spreader (12) and heated using a resistive ring heater (13). Interference images are obtained using a laser (14), combined with a beam splitter (15), a 10X or 4X objective (16) and the high-speed camera (8). T_{S} is measured using a T-type thermocouple.

Supplementary Note 1: Other Leidenfrost Studies. Potential reasons why other studies did not observe the trampolining motion of Leidenfrost droplets: We found that trampolining only occurs for droplets with Bo < 0.5, so that numerous studies investigating rather large droplets were not able to observe it. Furthermore, small droplets tend to mobilize spontaneously in the horizontal direction[1] quickly moving out of the field of view making an observation of Leidenfrost trampolining challenging. In numerous studies the droplets are strictly confined (in very small cavities of the size of the droplet) or immobilized via needles, which could also suppress the trampolining motion[2]. Some studies also investigate the Leidenfrost effect for highly viscous liquids, where we showed that high liquid viscosity suppresses Leidenfrost trampolining.

Supplementary Note 2: Surface Temperature Effect. The equilibrium vapor cushion thickness d_0 depends on the surface temperature T_S. In contrast, we found that once at the Leidenfrost state, the trampolining heights do not depend on T_S. This is the case since the achievable trampolining height is not directly related to the equilibrium vapor cushion thickness d_0. Once the droplet is trampolining, we are no longer in an equilibrium situation. Here it is the competition between the momentum gain during an interaction between the droplet and the hot surface that competes with the momentum loss of a droplet during the flight due to viscous dissipation that governs the achievable trampolining height. Based on our experiments we conclude that this competition is not substantially affected by the surface temperature.

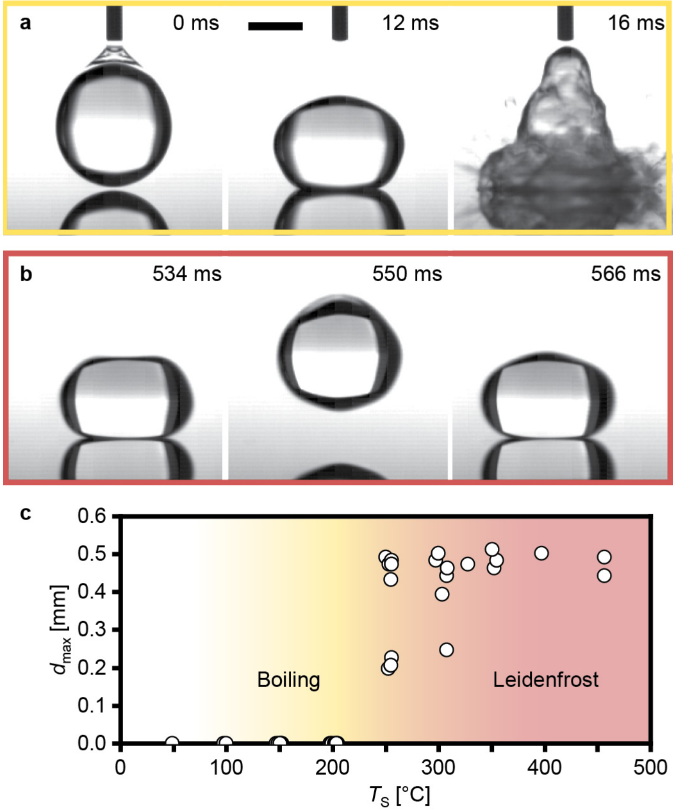

Supplementary Fig. 2 | The effect of surface temperature on Leidenfrost trampolining. **a**, Side-view photographs of a water droplet that boils when gently deposited on a flat silicon wafer heated to a temperature of T_S = 203 °C (Supplementary Movie 4). **b**, Side-view photographs of a water droplet showing Leidenfrost trampolining after being gently deposited on the same silicon wafer heated to T_S = 250 °C. **c**, Maximum gap height during a jump, d_{max}, as a function of T_S. While for T_S up to ≈ 200 °C the water evaporates or boils when placed on the hot surface, for T_S ≈ 250 °C and above Leidenfrost trampolining occurs. n = 47 individual experiments. Time-zero is when the droplet detaches from the needle. d_{max} is evaluated for the jump at t = 0.5 s. The initial droplet radius is r_i = 1.07 ± 0.03 mm (mean ± standard deviation) and the initial Bond number is Bo_i = 0.18. The scale bar of 1 mm applies to all images. Gentle deposition means here (in contrast to all other data), that the droplet either did not jump in the first oscillation after detachment from the needle or that it jumped by less than 0.05 mm (i.e. $d_{max,first} \leq$ 0.05 mm). Over the observation time of 0.5 s passing between droplet deposition and measurement of d_{max}, the instantaneous droplet radius, r, is practically constant. Source data are provided as a Source Data file.

Supplementary Note 3: Leidenfrost Modelling. We use a simple model to capture the fundamental physics of the Leidenfrost effect as proposed in Ref. [3]. For this model, we assume the following: The droplet shape can be approximated by a prismatic slab with a rectangular cross section with a height H and a width of $2W$. The droplet rests on a vapor cushion with an equilibrium thickness, d_0, which is constant over the entire droplet length. The gravitational force of the droplet is balanced by a pressure force. The pressure force arises from the pressure built up P under the droplet due to the flow of vapor escaping under the droplet that exceeds the environmental pressure, P_{env}. The flow of vapor is quasi-steady and developed with a parabolic profile subject to no-slip boundary conditions towards the droplet bottom interface and the hot substrate. The vapor is a Newtonian fluid. The flow under the droplet is symmetric along the channel length from the center of the droplet towards the edge. The liquid of the droplet is at rest and isothermal at a liquid temperature T_L. The temperature of the hot substrate is isothermal at T_S and well above the Leidenfrost temperature. At the interface between liquid and vapor the heat conducted through the vapor is balanced by a steady liquid evaporation.

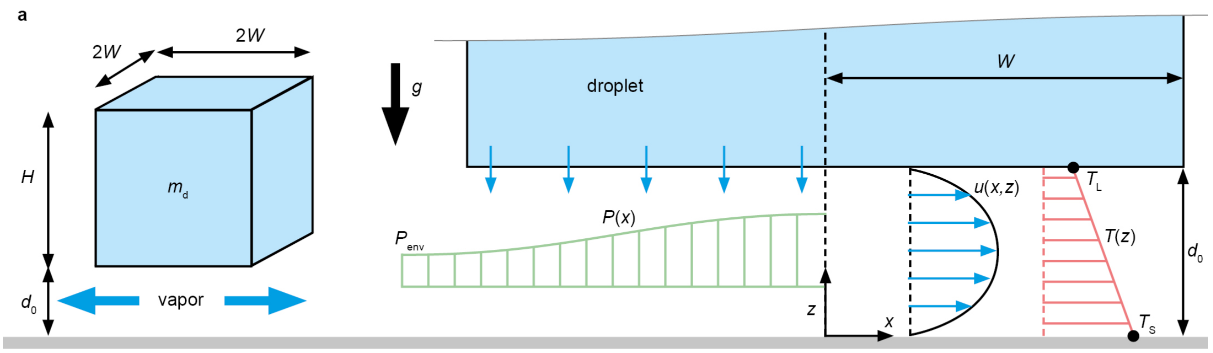

Supplementary Fig. 3 | Leidenfrost droplet modelling based on Ref. [3]. **a**, The droplet is assumed to be a cube of width $2W$, height H and hovering on a vapor cushion of thickness, d_0. **b**, The draining vapor takes a parabolic flow profile, $u(x, z)$. The temperature distribution in the vapor cushion is linear between the hot surface at T_S and the saturated liquid T_L. The pressure in the vapor cushion $P(x)$ is maximum at the center of the droplet and reduces along the x coordinate toward the edge down to the environmental pressure, P_{env}.

Based on these assumptions, we obtain for the vapor velocity

$$u = u_m\, 6\, z\, d_0^{-1}\, (1 - z\, d_0^{-1}),$$

where z is the vertical coordinate inside the vapor cushion starting with $z = 0$ at the hot substrate, u_m is the average vapor velocity, which is a function of the channel length x. Further, since we assume a steady state, we obtain a linear temperature distribution along the vapor cushion thickness, such that

$$T(z) = (1 - z\, d_0^{-1})\, (T_S - T_L) + T_L.$$

The governing equations of mass conservation, momentum balance and energy balance for the vapor flow simplify to

$$d_0\, (\mathrm{d}u_m / \mathrm{d}x) = a_v\, \mathrm{Ja}\, d_0^{-1}, \text{ (Eq. 1)}$$

$1.2\,\rho_v\,d_0\,(\mathrm{d}u_m^2 / \mathrm{d}x) = -\,d_0\,(\mathrm{d}P / \mathrm{d}x) - 12\,\mu_v\,u_m\,d_0^{-1}$ (Eq. 2)

where the Jakob number is defined as $\mathrm{Ja} = c_{P,v}\,(T_S - T_L)\,H_{lv}^{-1}$, with $c_{p,v}$ and H_{lv} being the isobaric heat capacity and the enthalpy of vaporization, respectively. $a_v = \kappa_v\,\rho_v^{-1}\,c_{P,v}^{-1}$ is the thermal diffusivity of vapor, with κ_v, and ρ_v being the thermal conductivity and the density, respectively. μ_v is the dynamic viscosity of vapor and P is the pressure in the vapor.

(Eq. 1) can be rewritten as

$u_m = a_v\,\mathrm{Ja}\,x\,d_0^{-2}$ (Eq. 3)

Inserting the expression (Eq. 3) into (Eq. 2) and integrating once, we obtain

$P(x) - P_{env} = 6\,\mu_v\,a_v\,\mathrm{Ja}\,W^2\,d_0^{-4}\,(1 + 0.2\,\mathrm{Ja}\,\mathrm{Pr}^{-1})\,[1 - (x / W)^2]$ (Eq. 4)

where the Prandtl number is defined as $\mathrm{Pr} = c_{P,v}\,\mu_v\,\kappa_v^{-1}$.

To obtain the effective pressure force per depth F_P' we integrate (Eq. 4) once along the entire channel length in x direction from $-W$ to $+W$:

$F_P' = \int_{-W}^{W}\,[P(x) - P_{env}]\,\mathrm{d}x = 8\,\mu_V\,a_V\,\mathrm{Ja}\,W^3\,d_0^{-4}\,(1 + 0.2\,\mathrm{Ja}\,\mathrm{Pr}^{-1})$

Based on the assumed vertical balance between pressure force, F_P', and gravitational force, F_G', we obtain in a force balance per depth:

$F_P' = 8\,\mu_v\,a_v\,\mathrm{Ja}\,W^3\,d_0^{-4}\,(1 + 0.2\,\mathrm{Ja}\,\mathrm{Pr}^{-1}) = 2\,(\rho_l - \rho_v)\,g\,H\,W = F_G'$ (Eq. 5)

We can solve (Eq. 5) for d_0 to obtain

$d_0 = [4\,\mu_v\,a_v\,\mathrm{Ja}\,W^2\,(1 + 0.2\,\mathrm{Ja}\,\mathrm{Pr}^{-1}) / ((\rho_l - \rho_v)\,g\,H)]^{1/4}$

For droplets with a radius less than the capillary length it is plausible to assume that the droplet is cubical so that $H = 2W$, and $W = (V_d / 8)^{1/3} = (\pi / 6)^{1/3}\,r = 0.81\,r$ (using $V_d = 4/3\,\pi\,r^3$) so that

$d_0 = [1.62\,\mu_v\,a_v\,\mathrm{Ja}\,r\,(1 + 0.2\,\mathrm{Ja}\,\mathrm{Pr}^{-1}) / ((\rho_l - \rho_v)\,g)]^{1/4}$

The effective overpressure is

$\Delta P = F_P' / (2W) = 4\,\mu_v\,a_v\,\mathrm{Ja}\,W^2\,d^{-4}\,(1 + 0.2\,\mathrm{Ja}\,\mathrm{Pr}^{-1})$.

Assuming $W = 0.81\,r$ we obtain the main text expression for the effective overpressure as

$\Delta P = 2.6\,\mu_v\,a_v\,\mathrm{Ja}\,r^2\,d^{-4}\,(1 + 0.2\,\mathrm{Ja}\,\mathrm{Pr}^{-1})$.

Supplementary Note 4: Overpressure Generation. Based on the theoretical treatment of the Leidenfrost flow in equilibrium (see Supplementary Text on Leidenfrost Modelling), the force due to gravity of the droplet is compensated by an equal but opposing pressure force that stems from the vapor draining under the droplet. For a given surface temperature, droplet size and liquid, the vapor cushion thickness is determined based on this equilibrium of forces. Any perturbation on the droplet bottom surface disturb this Leidenfrost equilibrium. Assuming a volume conserving perturbation with a square wave shape, the vapor cushion thickness will be increased in some regions under the droplet and reduced in other areas, as sketched in Supplementary Fig. 4a. The pressure force scales with the effective overpressure under the droplet, while the effective overpressure under the droplet scales non-linearly with the vapor cushion thickness, d. Effectively, the pressure force is proportional to d^{-4}. Therefore, a perturbation—hereafter termed ripple, which has an amplitude δ—superimposed over the equilibrium vapor layer thickness (within our simplifications) always translates into an increase of the effective overpressure, as shown in Supplementary Fig. 4b. The effective overpressure increase is computed as the ratio of the overpressure of the deformed droplet shape with a volume conserving ripple, $[\Delta P(d_0 - \delta) + \Delta P(d_0 + \delta)] / 2$, divided by the effective overpressure for the undeformed cubic droplet shape, $\Delta P(d_0)$. Inserting all variables (see Supplementary Text on Leidenfrost Modelling) we find that $[\Delta P(d_0 - \delta) + \Delta P(d_0 + \delta)] / [2\ \Delta P(d_0)] = 0.5\ \{[1 - (\delta / d_0)]^{-4} + [1 + (\delta / d_0)]^{-4}\}$, which shows that the overpressure gain is a general feature of the studied system that only depends on the relative ripple amplitude (δ / d_0). Consequently, as a ripple deforms the droplet bottom interface, the pressure force momentarily exceeds the force due to gravity, disturbing the Leidenfrost equilibrium. In summary, ripples—or more generally, perturbations—disturb the Leidenfrost equilibrium and cause an overpressure due to the non-linear dependence between vapor cushion thickness and pressure force.

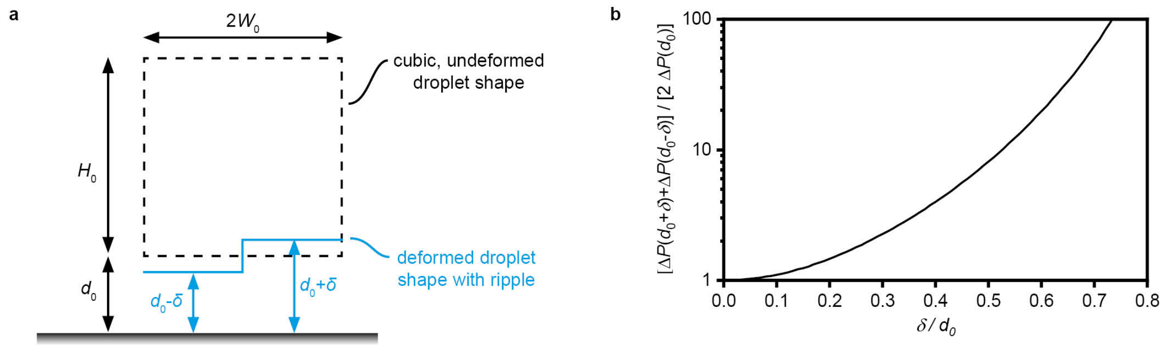

Supplementary Fig. 4 | Modeling the overpressure generation under the droplet due to an idealized perturbation. a, Undeformed droplet shape modeled as a cube (height H_0, width $2W_0$, depth $2W_0$, black dashed line) with a vapor cushion thickness that equals the equilibrium vapor cushion thickness ($d = d_0$), and deformed droplet shape (blue line) where the vapor cushion thickness is reduced by the perturbation amplitude δ over half the droplet width ($d = d_0 - \delta$), while it is increased over the other half of the droplet width ($d = d_0 + \delta$), thereby conserving the total droplet volume. **b**, The effective overpressure of the perturbed droplet shape, $[\Delta P(d_0 - \delta) + \Delta P(d_0 + \delta)] / 2$, divided by the effective overpressure for the undeformed cubic droplet shape, $\Delta P(d_0)$, versus the perturbation amplitude, δ, divided by the equilibrium vapor cushion thickness, d_0. Inserting all variables we find $[\Delta P(d_0 - \delta) + \Delta P(d_0 + \delta)] / [2\ \Delta P(d_0)] = 0.5\ \{[1 - (\delta / d_0)]^{-4} + [1 + (\delta / d_0)]^{-4}\}$, which shows that the overpressure gain is a general feature that only depends on the relative perturbation amplitude (δ / d_0).

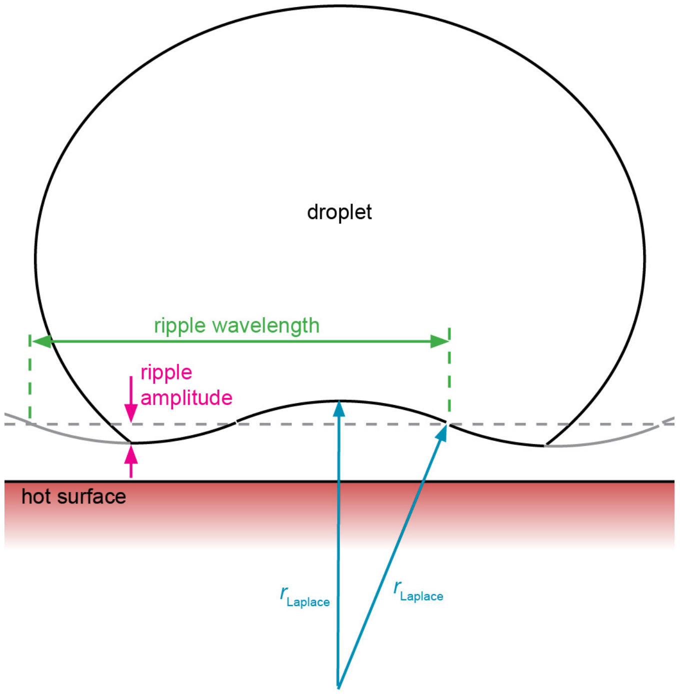

Supplementary Fig. 5 | Modified capillary number. The modified capillary number of the problem is defined as Ca* = $\Delta P_{viscous}$ / $\Delta P_{Laplace}$. The value of Ca* serves as a criterion to estimate whether the maximum viscous pressure of the draining vapor, $\Delta P_{viscous}$, can overcome the relevant resisting Laplace pressure of the droplet interface, $\Delta P_{Laplace}$, to create a ripple at the bottom of the droplet as shown (not to scale) in the sketch. The Laplace pressure is defined as $\Delta P_{Laplace} = 2\ \gamma\ r_{Laplace}^{-1}$. Based on experimental observations in Fig. 3 and Supplementary Fig. 8 and 10, the wavelength of the proposed ripple is approximated as r and the amplitude of the ripple is on the order of $d_0/4$. Consequently, $r_{Laplace} = 1/8\ (r^2 + d_0^2)\ d_0^{-1}$. When considering that $r >> d_0$, this simplifies to $r_{Laplace} = 1/8\ r^2\ d_0^{-1}$.

Supplementary Note 5: Mass-Spring-Damper Model. To shed light on how the overpressure can lead to trampolining, we can model a trampolining droplet as a mass-spring-damper (MSD) system with two individual masses and integrated heat transfer model. We found that in contrast to conventional droplet impact events that can be modeled with a MSD system with one degree of freedom (DOF) in the vertical direction (Ref. [4]), the Leidenfrost trampolining behavior (in the absence of direct liquid contact with the substrate) can be captured by modelling the droplet with two individual masses and therefore with a total of two DOFs, see Supplementary Fig. 6. For the model, we assume the droplet mass m_d to be equally divided into these two masses, so that $m_1 = m_2 = 0.5m_d$. This approach enables the representation of both the droplet properties (including damping and stiffness), and the vapor cushion dynamics. To implement the model, we assume a simplified droplet geometry maintaining all the main features of the involved physics (Ref. [3]). In the undeformed state at initialization of the simulation, the droplet is modelled as a cube with initial height H_0, and width $2W_0$, at an initial distance to the hot surface of d_0, see Supplementary Fig. 6. During the simulation, the droplet geometry transitions to a cuboid with square-shaped footprint with a variable edge length of $2W$ and a variable height of H. The two masses are connected via a non-linear spring and a linear damper. The current vertical positions of the center of the two masses are expressed with the variables z_1 and z_2, respectively. The equations of motion are as follows:

$$m_1 \frac{d^2 z_1}{dt^2} + c_2 \frac{dz_1}{dt} - c_2 \frac{dz_2}{dt} + F_k = -m_1 g + F_p$$

$$m_2 \frac{d^2 z_2}{dt^2} - c_2 \frac{dz_1}{dt} + c_2 \frac{dz_2}{dt} - F_k = -m_2 g$$

where m_1 and m_2 are the two masses, z_1 and z_2 are their vertical center of mass positions (see Supplementary Fig. 6), t is time, c_2 is the damping constant, F_k is the non-linear spring force, F_p is the pressure force resulting from the draining vapor lifting the droplet, and $g = 9.81$ m s^{-2} is the gravitational acceleration.

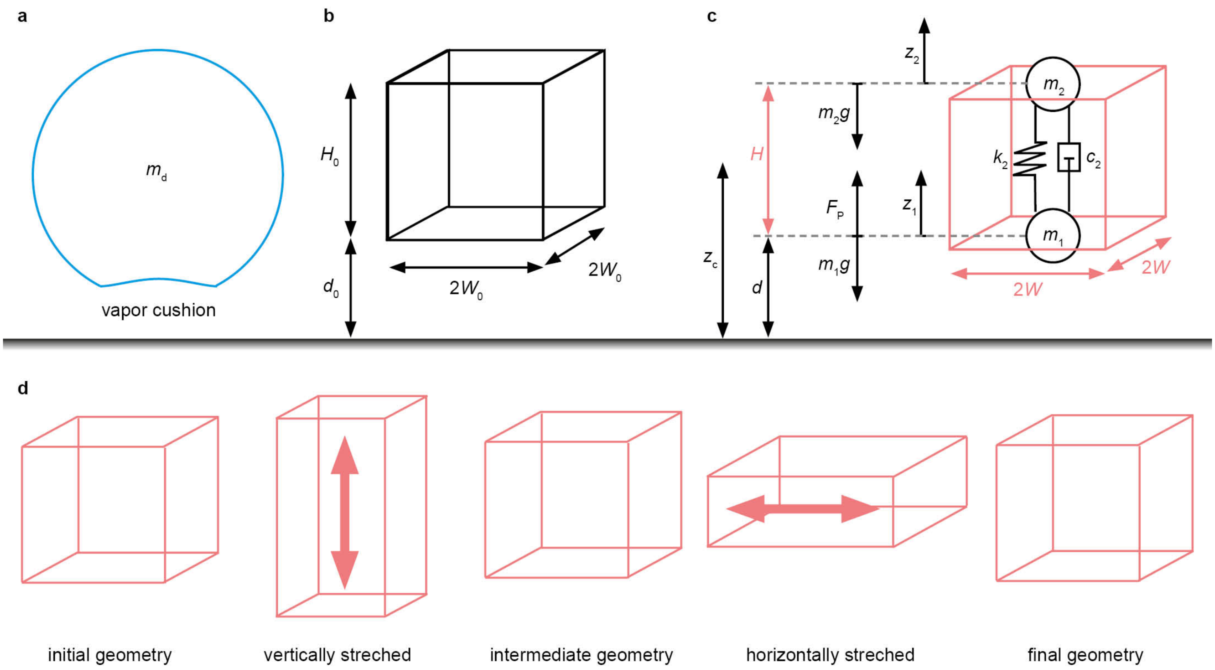

Supplementary Fig. 6 | Mass-spring-damper model of Leidenfrost droplet trampolining. **a**, The Leidenfrost droplet with a droplet mass m_d. **b**, The simplified droplet geometry similar to the model of Ref. [3]. In the undeformed state at initialization of the simulation, the droplet is modelled as a cube with initial height H_0, and width $2W_0$, at an initial vapor cushion thickness, d_0. **c**, Modelling the droplet motion via a mass-spring-damper system with two degrees of freedom in the vertical direction, with individual point masses m_1 and m_2, connected with a non-linear spring k_2 and a damper c_2, with the current vertical positions z_1, and z_2. During the simulation the geometry of the droplet (represented by red lines) transitions to a cuboid with square-shaped footprint with an edge length of $2W$ and a height of H. The vapor cushion thickness d varies over time. The droplet centroid position is defined as $z_c = d + 0.5\ H$. The pressure force F_p acts on mass 1, and forces due to gravity act on both masses. **d**, Example for an oscillation of the droplet in the simulation: Starting from the initial cubic geometry, the droplet transitions to a vertically stretched state, where the current height exceeds the initial height ($H > H_0$) and the current width is less than the initial width ($2W < 2W_0$), while conserving the total droplet volume, V_d. Through an intermediate cubic geometry, the droplet reaches a horizontally stretched state, where $H < H_0$ and $2W > 2W_0$, before ending in the final geometry that equals the initial geometry.

To run the simulation, we need to specify the damping constant, the spring force and the pressure force: The droplet damping constant, c_2, is estimated as $c_2 = 2\,\zeta\,(\gamma\, m_d)^{0.5}$ (Ref. [4]), where the damping ratio is $\zeta = 0.5\,(1-\varepsilon)\,(t_c\,/\,\tau)^{-1}$ (Ref. [5,6]). Here ε is the coefficient of restitution during a regular (non-trampolining) droplet impact event estimated to equal the maximum value of 0.91 (Ref. [7]) and $t_c\,/\,\tau$ is the contact time between the droplet and the surface, non-dimensionalized by the inertial-capillary time scale ($\tau = m_d^{0.5}\,\gamma^{-0.5}$), estimated to equal the so-called minimum contact time with $t_c\,/\,\tau = 1.09$ (Ref. [5,6]).

F_k is a force representing the stiffness of the droplet coming from its inherent surface tension, similar to a spring. For the derivation, we start from the surface energy of the droplet (assumed to be a cube as shown in Supplementary Fig. 6) $E_\gamma = 2\gamma\,(V_d\,H^{-1} + 2\,V_d^{0.5}\,H^{0.5})$ and differentiate it by the droplet height H to obtain the spring force in vertical direction as $F_k = -\mathrm{d}E_\gamma\,/\,\mathrm{d}H = 2\gamma\,(V_d\,H^{-2} - V_d^{0.5}\,H^{-0.5})$ (Ref. [4]), where $H = H_0 - z_1 + z_2$.

The pressure force is derived by integrating the overpressure under droplet resulting from the draining vapor (Eq. 4 in Leidenfrost Modelling) along the width of the droplet ranging from $-W$ to $+W$, and multiplying the result by the depth of the droplet of $2W$. We obtain a pressure force of $F_p = 16\,\Omega\,\mu_V\,a_V\,\mathrm{Ja}\,(1 + 0.2\,\mathrm{Ja}\,\mathrm{Pr}^{-1})\,(W\,/\,d)^4$, where $d = d_0 + z_1$ and $W = (0.25\,V_d\,H^{-1})^{0.5}$. It is important to note that the overpressure is obtained based on the classical Leidenfrost droplet analysis that assumes the vapor draining under the droplet to behave like a quasi-steady, quasi-developed incompressible flow in a one-dimensional channel (Ref. [3]). While this analysis is well suited for Leidenfrost droplets in equilibrium, we found that it requires adaptation for the more dynamic case of trampolining. This can be understood based on the relevant time scales of the problem: The duration that the droplet is in the proximity of the hot surface (meaning $d \approx 0.02$ mm) during trampolining is typically ≈ 10 ms in our experiments. This time is of the order of the vapor drainage time, that is defined as the channel length ($r \approx 1$ mm) divided by the speed of the draining vapor (typically between 0.1 m s^{-1} and 1 m s^{-1}) (Ref. [8]). Therefore, when the base of the droplet approaches the hot surface during trampolining, which is the case when $(\mathrm{d}z_1/\mathrm{d}t) < 0$, the channel flow of the draining vapor first has to develop and the pressure under the droplet has to charge over time, i.e. the flow is not immediately steady and developed. It is plausible that the effective pressure force is slightly released in this part of the event, while it is unaffected when the droplet departs from the surface and the flow had enough time to develop, which is the case for $(\mathrm{d}z_1/\mathrm{d}t) > 0$. We introduce this asymmetry into the pressure force via the prefactor Ω that we set to $\Omega = 0.81$ for $(\mathrm{d}z_1/\mathrm{d}t) < 0$ (that is when the droplet approaches the hot surface) and to $\Omega = 1$ for $(\mathrm{d}z_1/\mathrm{d}t) > 0$ (that is when the droplet departs from the hot surface). Furthermore, F_p takes into account the size variations of the square-shaped footprint of the droplet, since W changes during the simulation to account for droplet compression and with it changes in droplet height, H, while conserving the total droplet volume, V_d. While F_p is applied continuously to the droplet, it becomes practically negligible for $d >> 0$ since F_p is proportional to d^{-4}.

The simulation requires specification of the droplet volume V_d, the surface temperature T_S and the type of liquid (e.g. water). Here we chose $V_d = 5.6$ µL, $T_S = 300$ °C and water as a liquid to be comparable to the experiments in Fig. 1 and Fig. 2, and we set the liquid temperature to $T_L = 100$ °C.

With these inputs, the following constants for water are determined at saturation and a temperature of 100 °C (Ref. [9]): The liquid density $\rho_l = 958.35$ kg m^{-3}, the surface tension between liquid and vapor $\gamma = 0.05892$ N m^{-1}, the specific heat capacity of vapor $c_{p,v} = 2080.0$ J kg^{-1} K^{-1}, the dynamic viscosity of vapor $\mu_v = 12.232\ 10^{-6}$ Pa s, the specific enthalpy of vaporization $h_{lv} = 2256.4\ 10^3$ J kg^{-1}, the vapor density $\rho_v = 0.59817$ kg m^{-3}, the thermal conductivity of saturated vapor $\kappa_v = 0.02457$ W m^{-1} K^{-1}, the Jakob number $\mathrm{Ja} = c_{p,v}\,(T_S - T_L)\,h_{lv}^{-1}$, the thermal diffusivity of vapor $a_v = \kappa_v\,c_{p,v}^{-1}\,\rho_v^{-1}$, and the Prandtl number $\mathrm{Pr} = c_{p,v}\,\mu_v\,\kappa_v^{-1}$.

To initialize the simulation we compute the following initial values: The initial half-width of the cube base $W_0 = (1/8\ V_d)^{1/3}$, the initial height of the cube $H_0 = 2\ W_0$, the droplet mass $m_d = V_d\,\rho_l$, the masses of the first and the second mass $m_1 = m_2 = 0.5m_d$, the initial vapor cushion thickness $d_0 = [4\ \mu_v\ a_v\ \mathrm{Ja}\ W_0^2\ (1 + 0.2\ \mathrm{Ja\ Pr}^{-1})\ /\ (H_0\ g\ \rho_l)]^{1/4}$ resulting from equating the force due to gravity with the pressure force. The simulation is implemented and solved in MATLAB Simulink. It usually runs for a simulation time of 10 s with a time step of 0.00001 s.

Supplementary Fig. 7 shows the results. The model captures the main dynamics of Leidenfrost droplet trampolining. After the start of the simulation, the cubical droplet slightly compresses due to gravity. This small initial motion suffices to trigger the droplet to vibrate with increasing amplitude (represented by the oscillatory motion of the centroid position z_c) until it performs its first jump where the vapor cushion thickness d exceeds the equilibrium vapor cushion thickness. The amplitude of z_c (where z_c varies between 0.7 and 1.3 mm), the magnitude of d (reaching values of up to 0.4 mm), as well as the frequency of the vibrations match well with the experimental results (e.g. Figure 1). The simulation further allows to quantify the magnitude of the pressure force F_p as compared to the force due to gravity ($m_d\ g$), see Supplementary Fig. 7. We find that the ratio $F_p\ /\ (m_d\ g)$ can briefly exceed 50, which illustrates that the pressure force can overcome gravity and propel the droplet away from the hot surface. Interestingly, even when accounting for the compression effect of gravity onto the droplet body and reducing the initial perturbations (by initializing the droplet as a compressed cuboid with appropriate height and width), we observe a similar trampolining type-response since the system is inherently underdamped. Further, the simulation sheds light to the difficulty of large droplets to perform a trampolining motion. In experiments, we observed that large droplets do not behave similar to an elastic bouncing ball, but instead wobble with multiple uncoordinated modes of macroscopic droplet oscillation dissipating more kinetic energy, which is no longer available to the bouncing motion. In the simulation, this can be translated into a substantially higher effective damping response of the system similar to the addition of glycerol to the water droplet (Supplementary Fig. 12). To illustrate the effect of increased damping, we can show that for a doubled damping coefficient and otherwise unchanged conditions, no trampolining motion occurs. In summary, the created overpressure drives the underdamped Leidenfrost droplet (that can be modelled as a MSD system with two DOFs) into a trampolining motion by supplying momentum to the droplet as the droplet approaches the hot surface. Uncoordinated droplet motion (wobbling) due to increased droplet size and increased liquid viscosity due to the addition of glycerol dampens the dynamics (both in simulation and experiment).

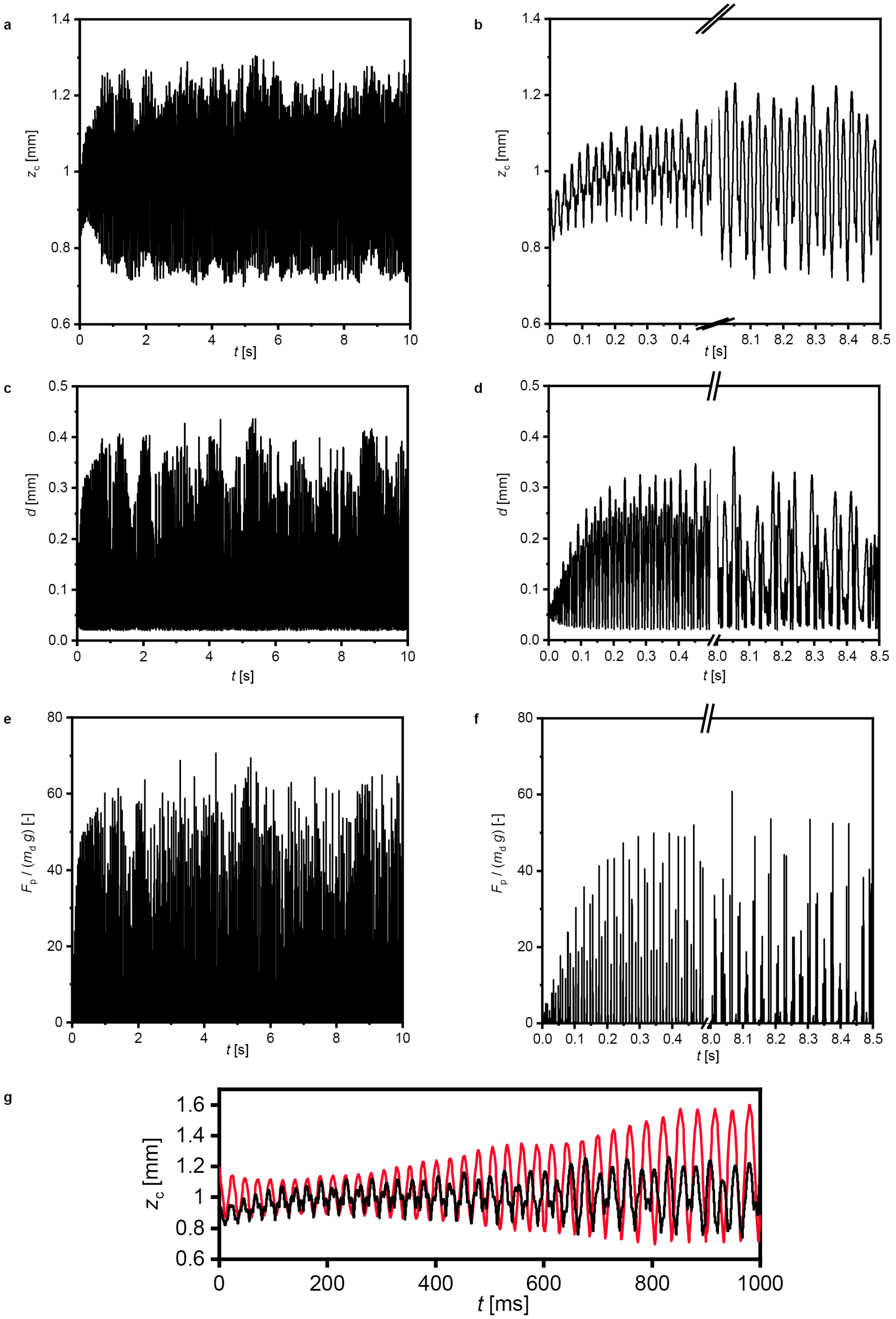

Supplementary Fig. 7 | Simulation results from the mass-spring-damper model showing, **a-b,** the droplet centroid position, z_c, **c-d,** the vapor cushion thickness, d, and, **e-f**, the ratio between pressure force, F_p, and force due to gravity, (m_d g), vs. time, t. **g**, Comparison of z_c as measured experimentally (red line, Fig. 1) and simulated (black line). Source data are provided as a Source Data file.

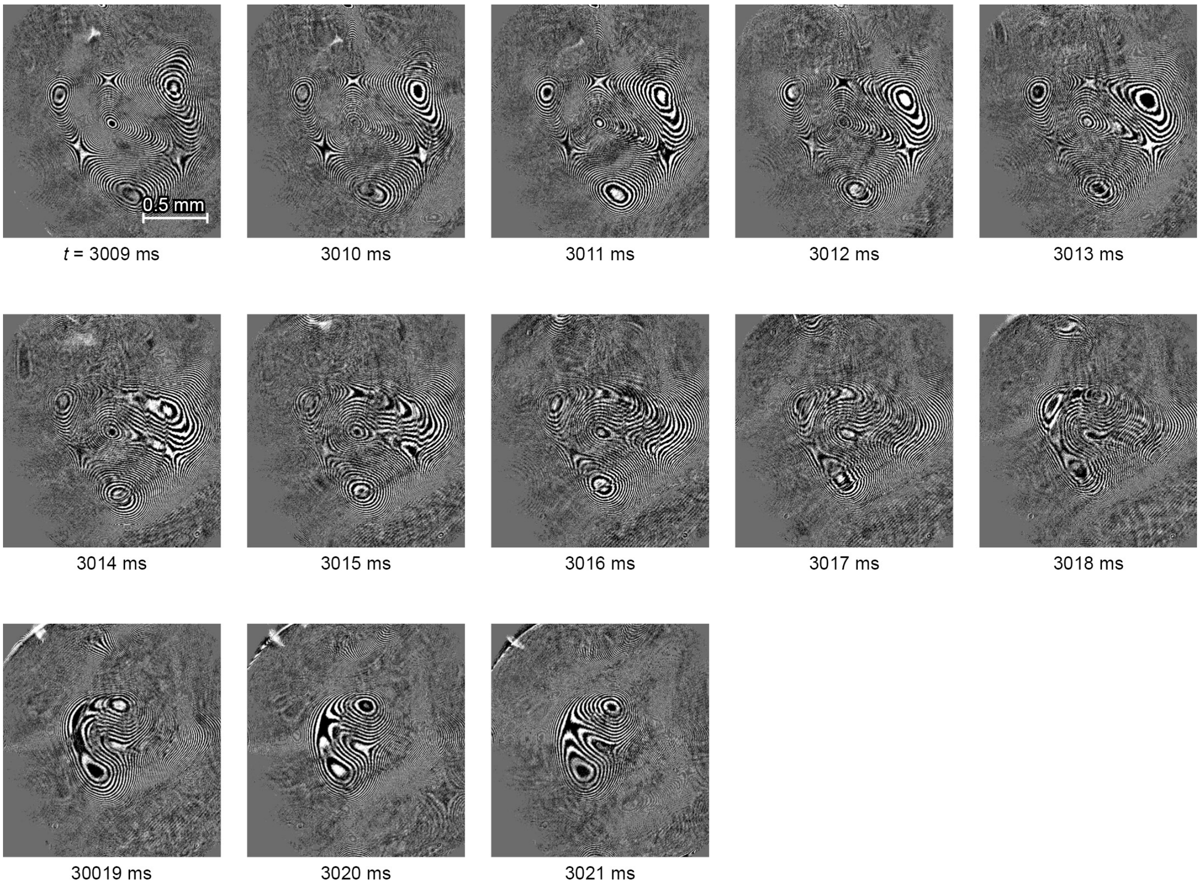

Supplementary Fig. 8 | Interferometric microscopy of a large Leidenfrost water droplet. Bottom-view interferometric images of relatively large (not trampolining) Leidenfrost droplet on a heated sapphire plate (T_S = 280 °C). The vapor pocket radius is l = 1.1 mm. Time-zero is the start of droplet observation (Supplementary Movie 5).

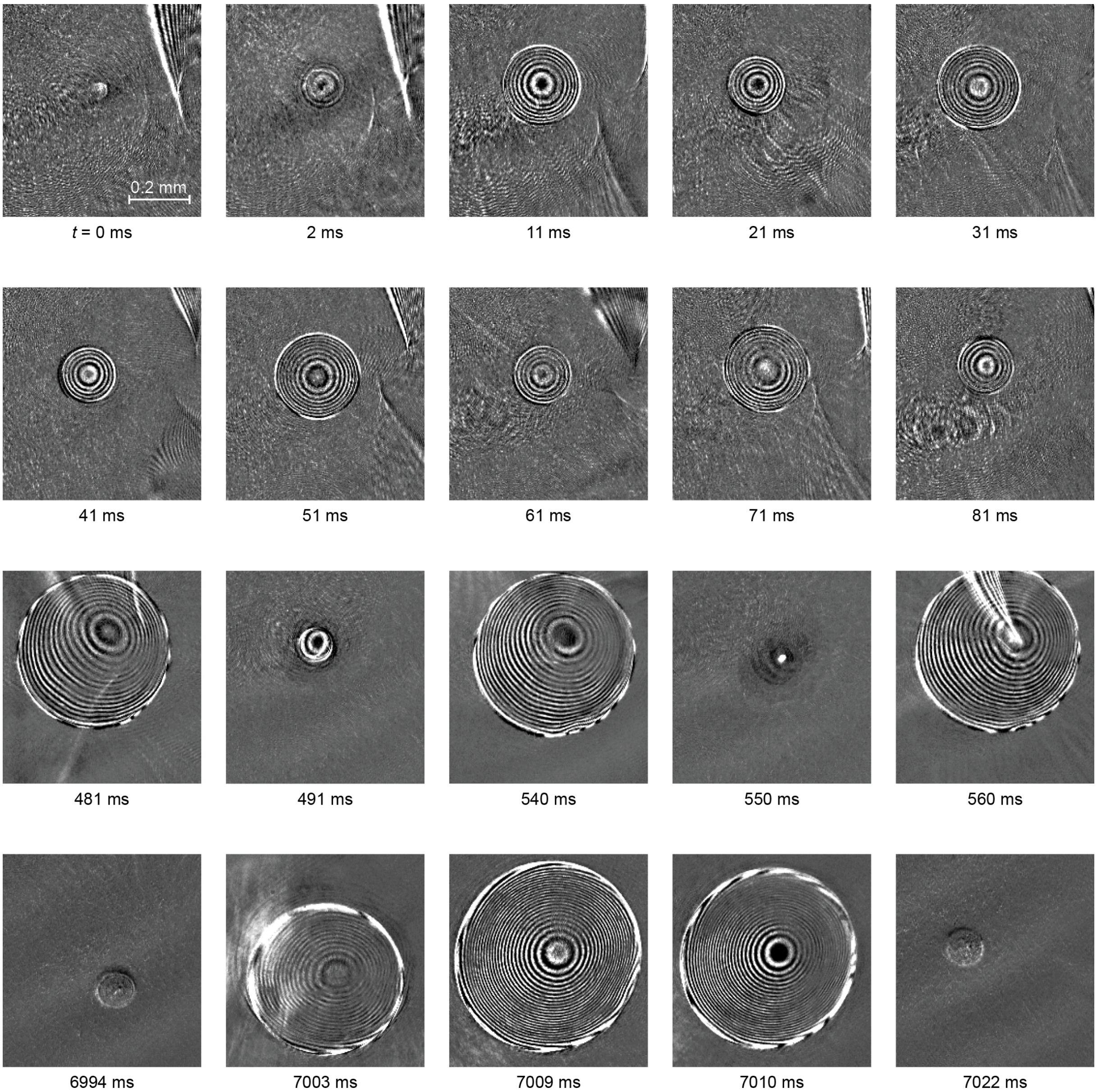

Supplementary Fig. 9 | Interference imaging of an intermediate-sized, trampolining droplet on a heated sapphire plate at T_S = 250 °C. Image sequence showing the gentle deposition of the droplet on the heated surface and the subsequent droplet dynamics. Time-zero is the moment the droplet is placed on the heated surface, while still remaining in contact with the deposition needle to immobilize the droplet. The successive frames show the maximum and minimum vapor pocket size during the oscillations and highlight how the droplet oscillates with increasing intensity up to the point of the first jump at t = 550 ms. The droplet is still trampolining 7 s after droplet deposition. The bottom row shows a full cycle of bouncing starting from no contact (first frame), impact and spreading (second frame), maximum spreading (third frame), receding (fourth frame) and jumping again (fifth frame). The initial droplet radius is $r_i \approx 1$ mm (Supplementary Movie 6).

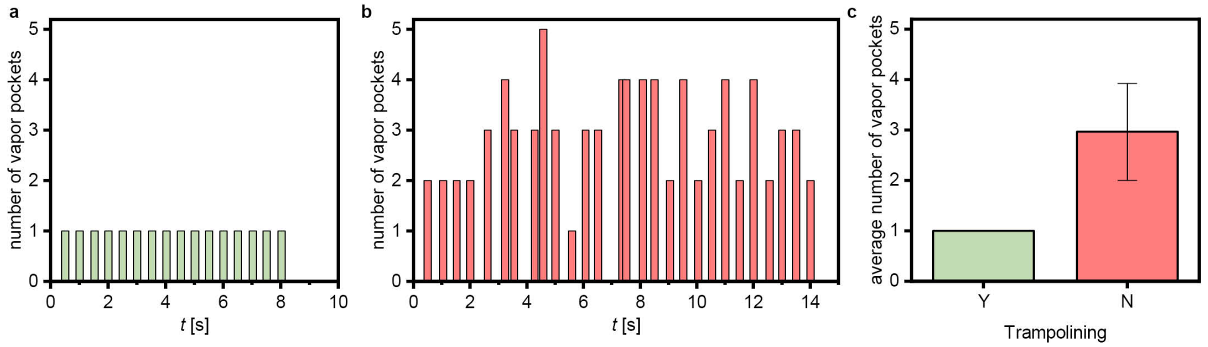

Supplementary Fig. 10 | Number of vapor pockets observed with interference imaging. a, Number of vapor pockets observed for an intermediate-sized, trampolining droplet on a heated sapphire plate at T_S = 250 °C (as shown in Supplementary Fig. 9). Time-zero is the moment the droplet is placed on the heated surface, while still remaining in contact with the deposition needle to immobilize the droplet. The initial droplet radius is $r_i \approx 1$ mm. **b**, Number of vapor pockets observed for a large Leidenfrost water droplet on a heated sapphire plate at T_S = 280 °C (as shown in Supplementary Fig. 8). The vapor pocket radius is l = 1.1 mm. Time-zero is the start of droplet observation. **c**, Average number of observed vapor pockets based on the data in **a** and **b**, for a droplet that is trampolining (Y = yes) and a droplet that is not trampolining (N = no). The error bar represents the standard deviation. n = 16 observations for the trampolining droplet and n = 28 observations for the not trampolining droplet. The number of vapor pockets is evaluated approximately every 0.5 s. Due to limited image contrast (as shown in Supplementary Fig. 8) and the effect of droplet motion (as shown in Supplementary Fig. 9) counting the number of vapor pockets is challenging especially for the non-trampolining droplets. Therefore, we cannot evaluate the number exactly every 0.5 s. Instead we search for a sufficiently clear image every 0.5 s that we use for the counting. As a result, there are slight deviations from the 0.5 s rhythm (see **b**). Source data are provided as a Source Data file.

Supplementary Note 6: Droplet Damping. There are different experimental observations that we relate to the effect of damping within the liquid droplet. We perform our most direct investigation on the effect of liquid damping by adding glycerol to the water droplets (thereby increasing the liquid viscosity, μ_l) and investigate the effect on the occurrence of Leidenfrost trampolining. From standard droplet impact experiments on liquid repellent surfaces it is known that increasing μ_l can result in rebound suppression in situations where a less viscid liquid shows rebound, which is associated with increased viscous losses during droplet deformation[10,11]. Energy transformed in viscous losses is no longer available as kinetic energy of the droplet and reduces the rebound abilities. In our mass-spring-damper model, viscous losses are represented by the linear damper between the two parts of the droplet (see Supplementary Text on Mass-Spring-Damper Model). Therefore, in the model, we can represent the addition of glycerol and the related increase in μ_l by an increase of the damper constant of the linear damper. To test this relation, we doubled the damper constant and found that, indeed, trampolining ceases to exist in the model for otherwise unchanged conditions.

Droplet wobbling is another aspect that translates effectively into a reduction of available kinetic energy for the droplet to perform the vertical trampolining motion. Experimentally, we find that intermediate-sized droplets do not wobble. They behave like an elastic ball. In contrast, large droplets wobble. This can be observed both from the side-view perspective and from the bottom-view perspective with interference imaging. Especially from the bottom-view images we see that there are various overlaying, uncoordinated droplet oscillations on the surface of large droplets (see Fig. 3). The energy required to perform the wobbling motion and the additional viscous losses related to wobbling are no longer available for the vertical trampolining motion, which effectively increases the damping of the droplet against a vertical trampolining motion. This has a similar effect as increasing μ_l, trampolining ceases to exist for wobbling droplets. A simple way to study why large droplets tend to wobble more than intermediate-sized droplets is to compute the droplet-size-dependent oscillation mode decay time given as $t_m = \rho_l\, r^2\, [\mu_L\, (m_{nbr} - 1)\, (2m_{nbr} + 1)]^{-1}$ for freely oscillating droplets, where μ_l is the liquid viscosity and m_{nbr} is an integer representing the oscillation mode number of the various vibration modes of the liquid droplet[12,13]. It is important to note that the mode decay time is given for freely oscillating droplets, which results in substantially higher decay times than what should be expected for the droplets in our experiments that are hovering above a surface and thereby experience an additional damping effect due to the deformation and interactions with the vapor cushion. Nevertheless, the analysis can guide our understanding of the droplet size effect on persisting oscillations and wobbling. In Supplementary Fig. 11 we show t_m vs. the Bond number, Bo, for different m_{nbr} and μ_l.

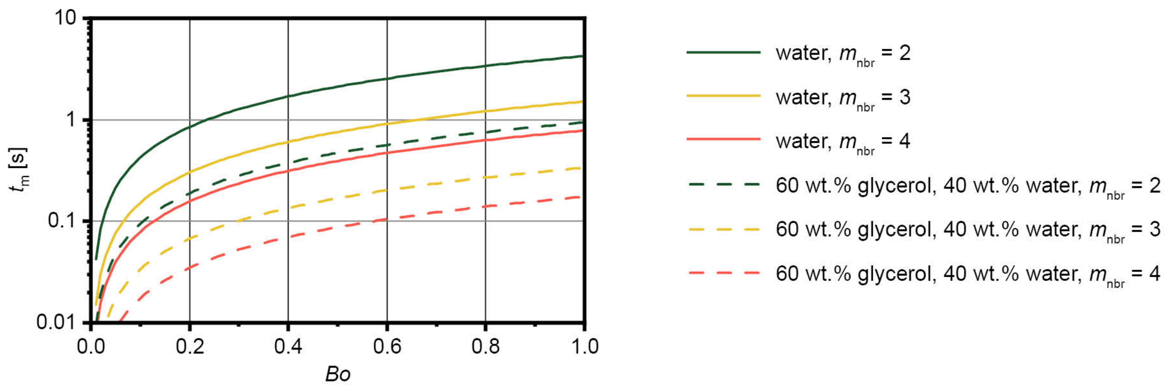

Supplementary Fig. 11 | Mode decay time, t_m, for freely oscillating droplets as a function of the droplet Bond number, Bo, the mode number, m_{nbr}, and the liquid composition (either pure water or an aqueous solution with 60 wt.% glycerol). Oscillations with higher m_{nbr}, have shorter decay times, i.e. decay faster. Adding glycerol to the water and thereby increasing the liquid viscosity, μ_{l}, reduces the decay time. For intermediate-sized droplets (Bo $\approx$ 0.1) t_m ranges between 0.01 and 1 s, while for large droplets (Bo $\approx$ 1) t_m ranges between 0.1 and 10 s. Source data are provided as a Source Data file.

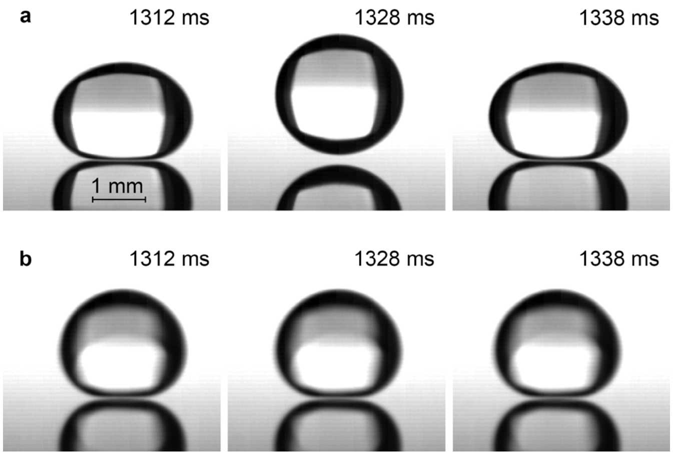

Supplementary Fig. 12 | Effect of liquid viscosity on Leidenfrost droplet trampolining. a, A droplet consisting of 70 wt.% water mixed with 30 wt.% glycerol showing Leidenfrost trampolining on a flat silicon wafer (Supplementary Movie 8) (T_S = 303 °C). **b**, A droplet consisting of 40 wt.% water mixed with 60 wt.% glycerol hovering in a Leidenfrost state on the same surface as in **a** without showing Leidenfrost trampolining (Supplementary Movie 9) (T_S = 301 °C). The scale bar in **a** applies to all images. Time-zero is the moment the droplet detaches from the needle.

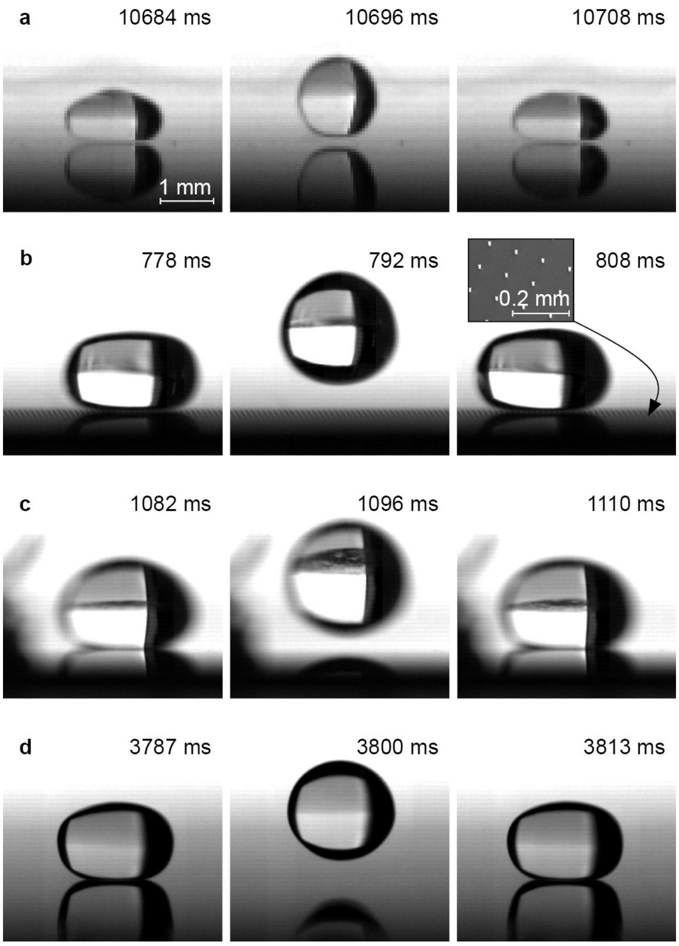

Supplementary Fig. 13 | Robustness of Leidenfrost trampolining. a, A droplet of liquid nitrogen trampolining on a concave silicon surface. The surface is at room temperature with T_S = 23 °C (Supplementary Movie 10). **b-d**, Water droplets showing Leidenfrost trampolining on a micro-textured silicon surface heated to a surface temperature of T_S = 394 °C, **b** (Supplementary Movie 11); on a flat, hydrophobic silicon surface heated to T_S = 204 °C, **c** (Supplementary Movie 12); and on a polished, concave aluminum surface heated to T_S = 303 °C, **d** (Supplementary Movie 13). The inset in **b** shows a micrograph of the silicon micro-pillar surface. The scale bar in **a** applies to all images. For **a**, time-zero is the start of droplet observation. For **b-d**, time-zero is the time the droplet detaches from the needle.

Supplementary References

1. Bouillant, A. *et al.* Leidenfrost wheels. *Nat. Phys.* **14**, 1188–1192 (2018).
2. Ma, X. & Burton, J. C. Self-organized oscillations of Leidenfrost drops. *J. Fluid Mech.* **846**, 263–291 (2018).
3. Arpaci, V. S. & Larsen, P. S. *Convection Heat Transfer*. (1984).
4. Schutzius, T. M. *et al.* Spontaneous droplet trampolining on rigid superhydrophobic surfaces. *Nature* **527**, 82–85 (2015).
5. Bird, J. C., Dhiman, R., Kwon, H.-M. & Varanasi, K. K. Reducing the contact time of a bouncing drop. *Nature* **503**, 385–388 (2013).
6. Liu, Y. *et al.* Pancake bouncing on superhydrophobic surfaces. *Nat Phys* **10**, 515–519 (2014).
7. Richard, D. & Quere, D. Bouncing water drops. *Europhys. Lett.* **50**, 769–775 (2000).
8. Biance, A. L., Clanet, C. & Quere, D. Leidenfrost drops. *Phys. Fluids* **15**, 1632–1637 (2003).
9. *CRC Handbook of Chemistry and Physics*. (CRC Press, 2005).
10. Mao, T., Kuhn, D. C. S. & Tran, H. Spread and Rebound of Liquid Droplets upon Impact on Flat Surfaces. *AIChE J.* **43**, 2169–2179 (1997).
11. Graeber, G., Martin Kieliger, O. B., Schutzius, T. M. & Poulikakos, D. 3D-Printed Surface Architecture Enhancing Superhydrophobicity and Viscous Droplet Repellency. *ACS Appl. Mater. Interfaces* **10**, 43275–43281 (2018).
12. Lamb, H. *Hydrodynamics*. (Cambridge University Press, 1916).
13. Becker, E., Hiller, W. J. & Kowalewski, T. A. Experimental and theoretical investigation of large-amplitude oscillations of liquid droplets. *J. Fluid Mech.* **231**, 189–210 (1991).
