# Supplementary material for: Leidenfrost droplet trampolining
Source: Nat Commun. 2021 Mar 19;12:1727. doi: 10.1038/s41467-021-21981-z (PMC7979863; doi:10.1038/s41467-021-21981-z)
Supplement: Supplementary file 2 — Descriptions of Additional Supplementary Files [file 41467_2021_21981_MOESM2_ESM.pdf]

Descriptions of Additional Supplementary Files

Supplementary Movie 1

Description: Leidenfrost droplet trampolining. Sideview video of a water droplet showing Leidenfrost trampolining after being gently deposited on a flat silicon wafer heated to a temperature of TS = 251 °C.

Supplementary Movie 2

Description: Effect of droplet size, Side-view, long-term video of a water droplet showing Leidenfrost trampolining after being gently deposited on a concave silicon wafer heated to a temperature of TS = 302 °C.

Supplementary Movie 3

Description: Leidenfrost trampolining with acetone, ethanol and IPA. Side-view video of acetone, ethanol and isopropyl alcohol (IPA) droplets showing Leidenfrost trampolining after being gently deposited on a concave silicon wafer heated to a temperature of TS = 300 °C.

Supplementary Movie 4

Description: Effect of surface temperature. Side-view video of a water droplet that starts to boil after being gently deposited on a flat silicon wafer heated to a temperature of TS = 203 °C.

Supplementary Movie 5

Description: Effect of droplet size on droplet oscillations (large droplet). Bottom-view, interferometric microscopy video of a comparably large Leidenfrost water droplet on a sapphire plate heated to TS = 280 °C.

Supplementary Movie 6

Description: Effect of droplet size on droplet oscillations (small droplet). Bottom-view, interferometric microscopy video of a comparably small water droplet showing Leidenfrost trampolining after being deposited on a sapphire plate heated to a temperature of TS = 250 °C.

Supplementary Movie 7

Description: Effect of droplet size on droplet oscillations (transition from large to small droplet). Synchronized side-view and bottom-view video of a large water droplet being gently deposited on a concave sapphire surface heated to TS = 278 °C. Initially, the droplet is too large to trampoline (Bo = 0.9). Over time it shrinks and finally starts Leidenfrost trampolining (Bo = 0.3). Time-zero is the start of droplet observation.

Supplementary Movie 8

Description: Effect of droplet viscosity (low viscosity). Side-view video of a droplet consisting of 70 wt.% water mixed with 30 wt.% glycerol showing Leidenfrost trampolining after being gently deposited on a flat silicon wafer heated to a temperature of TS = 303 °C.

Supplementary Movie 9

Description: Effect of droplet viscosity (high viscosity). Side-view video of a droplet consisting of 40 wt.% water mixed with 60 wt.% glycerol not showing Leidenfrost trampolining after being gently deposited on a flat silicon wafer heated to a temperature of TS = 301 °C.

Supplementary Movie 10

Description: Leidenfrost trampolining with liquid nitrogen. Side-view video of a droplet of liquid nitrogen showing Leidenfrost trampolining after being gently deposited on a concave silicon wafer at a surface temperature of TS = 23 °C. Supplementary Video 11 | Effect of surface texture | Side-view video of a water droplet showing Leidenfrost trampolining after being gently deposited on a micro-textured silicon surface heated to a surface temperature of TS = 394 °C.

Supplementary Movie 12

Description: Effect of surface chemistry. Side-view video of a water droplet showing Leidenfrost trampolining after being gently deposited on a flat, hydrophobic silicon surface heated to TS = 204 °C.

Supplementary Movie 13

Description: Effect of surface material. Side-view video of a water droplet showing Leidenfrost trampolining after being gently deposited on a polished, concave aluminum surface heated to TS = 303 °C.
